# Predictors of working beyond retirement in older workers with and without a chronic disease - results from data linkage of Dutch questionnaire and registry data

**DOI:** 10.1186/s12889-018-5151-0

**Published:** 2018-02-17

**Authors:** Astrid de Wind, Micky Scharn, Goedele A. Geuskens, Allard J. van der Beek, Cécile R. L. Boot

**Affiliations:** 1 Department of Public and Occupational Health, Amsterdam Public Health research institute, VU University Medical Center, Van der Boechorststraat 7, 1081 BT Amsterdam, The Netherlands; 2 Body@Work, Research Center on Physical Activity, Work and Health, TNO-VU/VUmc, Amsterdam, The Netherlands; 3 Netherlands Organisation for Applied Scientific Research TNO, Schipholweg 77-89, 2316 ZL Leiden, The Netherlands

**Keywords:** Longitudinal study, Prediction model, Big data, Ageing, Older workers, Retirees, Chronic disease, Working beyond retirement

## Abstract

**Background:**

An increasing number of retirees continue to work beyond retirement despite being eligible to retire. As the prevalence of chronic disease increases with age, working beyond retirement may go along with having a chronic disease. Working beyond retirement may be different for retirees with and without chronic disease. We aim to investigate whether demographic, socioeconomic and work characteristics, health and social factors predict working beyond retirement, in workers with and without a chronic disease.

**Methods:**

Employees aged 56–64 years were selected from the Study on Transitions in Employment, Ability and Motivation (*N* = 1125). Questionnaire data on demographic and work characteristics, health, social factors, and working beyond retirement were linked to registry data from Statistics Netherlands on socioeconomic characteristics. Separate prediction models were built for retirees with and without chronic disease using multivariate logistic regression analyses.

**Results:**

Workers without chronic disease were more likely to work beyond retirement compared to workers with chronic disease (27% vs 23%). In retirees with chronic disease, work and health factors predicted working beyond retirement, while in retirees without a chronic disease, work, health and social factors predicted working beyond retirement. In the final model for workers with chronic disease, healthcare work, better physical health, higher body height, lower physical load and no permanent contract were positively predictive of working beyond retirement. In the final model for workers without chronic disease, feeling full of life and being intensively physically active for > = 2 days per week were positively predictive of working beyond retirement; while manual labor, better recovery, and a partner who did not support working until the statutory retirement age, were negatively predictive of working beyond retirement.

**Conclusions:**

Work and health factors independently predicted working beyond retirement in workers with and without chronic disease, whereas social factors only did so among workers without chronic disease. Demographic and socioeconomic characteristics did not independently contribute to prediction of working beyond retirement in any group. As prediction of working beyond retirement was more difficult among workers with a chronic disease, future research is needed in this group.

## Background

Due to the ageing of the population, many governments have implemented measures that stimulate prolonged working and discourage early exit from the workforce. In the Netherlands, these policies are reflected in an increasing average age of leaving employment in the last decade, i.e. from 61 years in 2006 up to 64 years and 5 months in 2016 [1]. There is also an increasing proportion of retirees who actively engage in work activities while also receiving a pension, which is often referred to as working beyond retirement. In the Netherlands, the net labor participation rate among persons aged 65 to 75 years has doubled from 5.5% in 2003 to 11% in 2014 [2].

Previous public health and economic research showed that working beyond retirement has determinants in multiple domains, i.e. work characteristics, health, social factors and socioeconomic factors. To illustrate, within the domain of work characteristics, it was shown that employees working in manual labor and in healthcare were more likely to work beyond retirement than employees from other sectors. Employees having a permanent contract were less likely to work beyond retirement than employees with a temporary contract [3]. With regard to health, it was shown that employees with better physical health and those who are physically active for more than 30 min at least 2 days per week were more likely to work beyond retirement [3–5]. Within the social domain, the partner’s opinion on continued working played a role in previous research. It has also been shown that employees who are active in voluntary work were more likely to work beyond retirement. Previous research also reported that socioeconomic factors influence retirement timing. The larger the expected loss of income from retiring now rather than later, i.e. a higher option value, the lower the likelihood that people retire [6]. In another study, self-reported financial factors predicted working beyond retirement [4]. Altogether, these studies illustrate that the domains of work characteristics, health, social factors and socioeconomic factors play a role in working beyond retirement.

The changing age composition of the workforce is likely to have consequences for the health composition of the workforce as well. As the prevalence of chronic disease increases with age, it is likely that the workforce will consist of a larger proportion of persons suffering from chronic diseases. In 2013, 58% of the Dutch population aged 50 to 55 years, 66.2% of the population aged 55 to 65 years, and 72.7% of the population aged 65 to 75 years reports having at least one chronic disease [7]. Previous research showed that having a chronic disease may negatively impact participation in paid work. A systematic literature review showed that various chronic diseases were associated with exit from work via disability pension and unemployment [8]. However, because of reduced possibilities to leave work [9, 10], employees with chronic diseases who previously may have left the workforce may have to prolong their working lives. Thus, also workers who work beyond retirement may have a chronic disease.

As having a chronic disease may negatively impact participation in paid work, it is likely that workers with chronic diseases have specific needs and considerations with regard to working beyond retirement, as compared to the group without chronic diseases. Previous research on working until retirement may provide direction here. In a study among older workers with and without chronic diseases, those with chronic diseases benefited more from psychosocial resources at work, e.g. social support and autonomy, than those without chronic diseases [11]. Another study showed that autonomy at work mitigated the adverse effect of having a chronic disease upon early exit from the workforce [12]. These studies indicate that the domain of work characteristics may contribute to working beyond retirement differently for workers with chronic diseases than for workers without chronic diseases. It can be hypothesised that these differences between workers with and without chronic disease may also be present with regards to the domains of health, social factors and socioeconomic factors.

To summarise, previous research showed that working beyond retirement has determinants in the domains of work, health, and social and financial factors. In addition, several studies provided indications that older workers with chronic diseases have specific needs when it comes to participation in paid work. However, it is unclear whether the large and vulnerable group of older workers with chronic diseases is different from those without chronic disease when it comes to working beyond retirement. Therefore, the aim of the present study is to investigate whether demographic, socioeconomic and work characteristics, health and social factors predict working beyond retirement, in workers with and without a chronic disease.

## Methods

This study builds on a previous study of Scharn et al., that investigated whether demographic, socioeconomic and work characteristics, health, and social factors independently predict working beyond retirement [3]. The methods of the present study were integrated with those of this previous study to allow comparison.

### Datasets

The present study used data from the Study on Transitions in Employment, Ability and Motivation (STREAM). STREAM is a Dutch longitudinal study among 15,118 persons, including employees, self-employed persons, and persons without paid employment aged 45 to 64 years. Respondents participated in an internet panel and filled out yearly online questionnaires in 2010 to 2013, 2015 and 2016, and will do so in 2017 and 2018. The study population of STREAM has been extensively described previously [13].

Questionnaire data of STREAM were individually linked to register data of Statistics Netherlands i.e. the registers ‘integral personal income’, ‘capital equity of households in the Netherlands’ and ‘pension entitlements’ [14].

### Study population

**Fig. 1:**
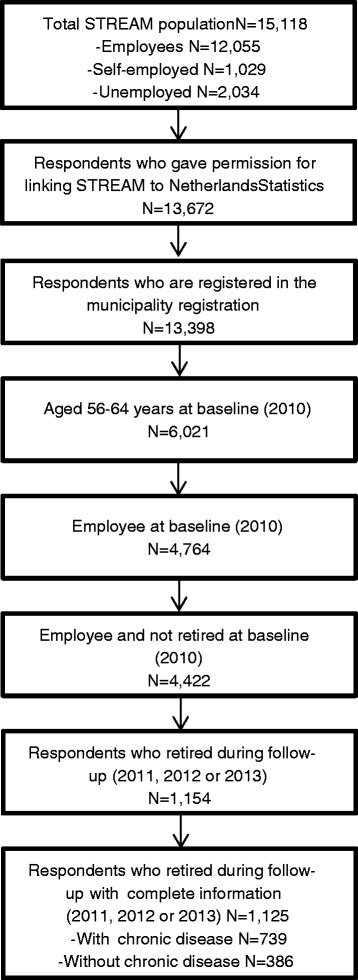
Flow of the study population

For the current study, we used the same inclusion and exclusion criteria as those used in the previous study on working beyond retirement [3]. Individuals were included in the analyses if they were an employee and aged 56 to 64 years at baseline and retired during follow-up. Persons needed to be an employee at baseline, as we were interested in the influence of job characteristics on working beyond retirement. Persons had to retire during follow-up because we wanted to compare retirees who worked beyond retirement with retirees who did not work beyond retirement. Retirement is defined as receiving a pension, either early retirement pension or old age pension (because someone reached the national statutory retirement age). We chose 56 years as the lower age limit as the proportion of employees who had retired, after one, two, or 3 years of follow-up strongly increased from this age onwards. Sixty-four years was the upper age limit, because this is the maximum age of persons included in STREAM. An additional inclusion criterion was permission for linking to the registers. A proportion of the STREAM respondents could not be linked to the registers, either because they could not be identified in the municipal registration (GBA) or because they did not have a social security number. Persons with missing information with regard to having a chronic disease were excluded. These inclusion and exclusion criteria resulted in a study population of 1125 persons (Fig. 1).

### Measurements

Independent variables in the domains of demographic and work characteristics, health and social factors were derived from the STREAM baseline questionnaire (2010). The selection of independent variables was based on our previous study reporting on a prediction model of working beyond retirement for older workers on the same population [3]. In line with the guidelines for prognostic prediction models, all variables that were hypothesised to be associated with working beyond retirement were selected in this previous study [15]. For the present study, it was necessary to limit the number of potential predictors, as the analyses were done for two separate subpopulations, i.e. employees with chronic diseases and employees without chronic diseases. Therefore, all variables that predicted working beyond retirement in the univariate analyses (*p*-value< 0.15) in our previous study were included as potential predictors in the present study, which is a much smaller set of predictors than the original set of predictors. Socioeconomic characteristics were derived from the registers ‘integral personal income’, ‘capital equity of households in the Netherlands’ and ‘pension entitlements’ from Statistics Netherlands (also 2010) [14]. The dependent variable, i.e. working beyond retirement, was derived from the follow-up questionnaires of STREAM (2011, 2012, 2013).

#### Chronic disease

To compare predictors of working beyond retirement between employees with and without chronic diseases, the study population was split into two separate subpopulations based on a question derived from STREAM: ‘Do you (currently) have one or more of the following chronic diseases, disorders or handicaps?’ This question had 13 answering options: problems with hands and arms, problems with legs and feet, problems with back and neck, severe headache or migraines, circulatory disorders, respiratory disorders, digestive disorders, diabetes mellitus, skin problems, psychological disorders, problems with hearing, epilepsy, life threatening illnesses, problems with vision, and other chronic diseases, disorders or handicaps. Those who indicated having at least one of these chronic diseases were classified as employees with chronic diseases and those who did not indicate any of these were classified as employees without chronic diseases.

#### Working beyond retirement

The dependent variable was working beyond retirement, defined as working while also receiving a pension, either early retirement pension, or old age pension, in 2011, 2012 or 2013. Information on employment status was derived from a question (‘In which situation are you currently?’), with, among others, response options (employee, retirement, early retirement). Those who indicated to be employed and in (early) retirement were considered as working beyond retirement. Those who work while also receiving a pension were compared to those who receive a pension, but do not participate in paid work (anymore).

#### Independent variables

In the domain of demographic characteristics, we included educational level. Educational level was measured using a question on the highest level of education completed with a diploma, and categorised into low (primary school, lower and intermediate secondary education or lower vocational training), intermediate (higher secondary education or intermediate vocational training), and high (higher vocational education or university).

Socioeconomic characteristics that we included were income, equity and option value. Due to skewed distributions, all socioeconomic characteristics were log transformed, and income and equity were categorised in quartiles. Income concerns the net personal income. Equity concerns the difference in monetary value between possessions and debts. Option value concerns the additional amount of income that is generated by prolonging work participation relative to immediate retirement. It was operationalised using two variables, i.e. the maximum amount of old age benefit a respondent could have accrued in 2010 subtracted by the total amount of old age benefit a respondent receives if he or she retires immediately.

Work characteristics that we included were profession (i.e. (semi-)skilled manual labor, transport and healthcare), having a permanent contract (yes/no), conducting supervisory tasks (yes/no), working with substances (yes/no), working in evening or night shifts (yes/no), physical and mental load, years of physically and mentally burdensome work, and procedural injustice. As previously described, all variables that predicted working beyond retirement in the univariate analyses (*p*-value< 0.15) in our previous study [3] were included as potential predictors in the present study. Following from this, only three sectors were included in the present study, i.e. working in (semi-)skilled manual labor, transport and healthcare. The reference group represents all other sectors. Physical load was measured using five items on force exertion, static load and vibrations (Cronbach’s alpha 0.86) based on the Netherlands Working Conditions Survey [16] and the Dutch Musculoskeletal Questionnaire [17]. An example item is ‘Do you work in awkward postures?’. Mental load was measured using slightly adjusted questions derived from NOVA-WEBA (Cronbach’s alpha 0.78) [18]. An example item is ‘Does your work require intensive thinking?’. The items on physical and mental load had a five-point answering scale ranging from ‘(almost) never’ to ‘always’. Thus, a higher score indicated higher load. Years of physically heavy work was expressed in 8 or more years in this type of work (vs less). Years of mentally heavy work was expressed in 16.5 or more years in this type of work (vs less). These variables were dichotomised based on content of the answering options, taking into account the frequency distribution (e.g., the median) to avoid nearly empty cells. Procedural injustice was measured using three items on equal treatment of employees and being taken seriously (Cronbach’s alpha 0.86) [19]. An example item is, ‘All employees are treated equally’. The original items had a five-point answering scale, ranging from ‘(almost) never’ to ‘always’, but in the present study, higher scores indicated higher procedural injustice.

Variables included in the health domain were general health, physical health, being full of life, worn out and fatigued, depressive symptoms, recovery and relaxation, pain in legs/ft (yes/no), body height (cm) and BMI (kg/m2), being physically active and being intensively physically active. General health was assessed using the following question from the Short-Form-12 Health Survey [20]: ‘How is your health in general?’ This question could be answered according to the following answering options: ‘excellent’, ‘very good’, ‘good’, ‘moderate’ and ‘poor’. Answering options ‘excellent’, ‘very good’ and ‘good’ were combined into ‘(very) good’, and the other options were combined into ‘moderate/poor’. Physical health was measured using the physical component summary scale (PCS) of the Short-Form-12 Health Survey [20]. The scale ranges from 0 to 100 (0 = worst and 100 = best possible health status). Being full of life, worn out and fatigue in the previous 4 weeks were assessed with three items derived from the Short-Form-36 Health Survey [21]. These items were converted into dichotomous variables. Response options ‘constantly’, ‘mostly’ and ‘often’ were categorised as ‘yes’ on the respective variable, and response options ‘sometimes’, ‘seldom’ and ‘never’ were categorised as ‘no’. Depressive symptoms were measured using a 10-item version of the Center for Epidemiologic Studies Depressions Scale (CES-D) that addresses depressive symptoms in the past week (Cronbach’s alpha 0.88) [22, 23]. These questions could be answered according to the following answering options; ‘seldom or never (less than 1 day)’, ‘sometimes or not a lot (1-2 days)’, ‘regularly (3–4 days)’ and ‘mostly or always (5–7 days)’, with a higher mean score indicating more depressive symptoms. Recovery and relaxation was measured by three questions based on the DISC-R (Cronbach’s alpha 0.71). An example item is ‘After a work day I don’t think about my work at all’. The items had a five-point answering scale ranging from ‘(almost) never’ to ‘always’, with a higher mean score indicating better recovery. Being physically active was expressed as being physically active at least 2 days per week for at least 30 min (vs less). Being intensively physically active was expressed as being intensively physically active at least 2 days per week for at least 20 min.

With regard to social factors, we included whether a partner had paid employment (yes/no), whether a partner was work disabled (yes/no), whether someone misses family activities due to work obligations (yes/no), and the attitude of the partner with regard to quitting work and continued employment. The attitude of the partner with regard to quitting work was analyzed according to the following categories: ‘no partner’, ‘very unpleasant/unpleasant’ and ‘neither pleasant nor unpleasant/pleasant’. The attitude of the partner with regard to continued employment was analyzed according to the following categories: ‘no partner’, ‘very unpleasant’, ‘unpleasant’, ‘neither pleasant nor unpleasant’, ‘pleasant’ and ‘very pleasant’.

### Analyses

The study population was split into two separate subpopulations, i.e. employees with chronic diseases and employees without chronic diseases. Descriptive statistics, i.e. means, standard deviations, frequencies, and percentages, were calculated to report on baseline characteristics of both subpopulations.

Building the prediction model took place in two steps, and each of these steps was done separately for the two subpopulations, i.e. employees with chronic diseases and employees without chronic diseases. First, multivariate logistic regression analyses with backward selection (*p* < 0.15) were conducted for every domain separately, i.e. socioeconomic, demographic and job characteristics, health, and social factors. Second, variables that were retained in the models from the domain analyses were included in a multivariate logistic regression analysis with backward selection. Variables with *p* < 0.05 were retained in the final multivariate model. Odds ratios (OR) and 95% confidence intervals (95% CI) were calculated to express the likelihood of working beyond retirement. The area under the curve (AUC) was calculated to express the accuracy of the prediction model. The accuracy depended on how well the test separates the group into employees who work beyond retirement and employees who do not work beyond retirement. The accuracy of the prediction model was considered to be good if AUC > .80, and fair if AUC > .70. Nagelkerke’s pseudo R2 was presented as a measure for the overall predictive ability. All statistical analyses were carried out using SPSS Statistic Version 20.

### Ethical issues

The Medical Ethical Committee of the VU University Medical Center Amsterdam declared that the Medical Research Involving Human Subjects Act does not apply to STREAM. The Medical Ethical Committee had no objection to the execution of this study. In the information for participants that accompanied the online questionnaire, it was emphasised that the privacy of participants was guaranteed, all answers to the questions were treated confidentially, and all data were stored in secured computer systems. By filling in the questionnaire, participants implicitly gave permission for the use of the data. Participants were explicitly asked for permission for linkage to register data of Statistics Netherlands, and those who did not give permission were not included in the analyses for the present study.

## Results

Table 1 shows the baseline characteristics of the employees with and without a chronic disease (*n* = 739 and 386, respectively). At baseline, employees were on average 61.3 years for both groups. Both groups contained more men than women, but the proportion of women was larger among the group with chronic diseases than among the group without chronic diseases (42.2% vs 36.8%). The employees with chronic disease more often had a low educational level (31.3% vs 26.9%) and less often a medium or high educational level (respectively, 33.7% vs 34.5% and 34.9% vs 38.6%). Of the employees with a chronic disease, 22.6% worked beyond retirement (*n* = 167). Of the employees without a chronic disease, 27.2% worked beyond retirement (*n* = 105).

**Table 1:** Baseline characteristics of employees with and without chronic disease (total population *N* = 1125)

	Unit, categories, range	Employees with chronic disease (*N* = 739)		Employees without chronic disease (*N* = 386)	
		Mean/N	SD/%	Mean/N	SD/%
Demographic characteristics					
Age	Years	61.29	1.87	61.33	1.97
Gender	Women	312	42.2%	142	36.8%
Educational level	Low	232	31.4%	104	26.9%
	Intermediate	249	33.7%	133	34.5%
	High	258	34.9%	149	38.6%
Socioeconomic characteristics					
Option value	Ln	6.52	3.05	6.64	3.12
Income	Q1 (0–25%)	312	42.2%	157	40.7%
	Q2 (26–50%)	79	10.7%	32	8.3%
	Q3 (51–75%)	192	26.0%	87	22.5%
	Q4 (76–100%)	156	21.1%	110	28.5%
Quartile equity	Q1 (0–25%)	186	38.7%	103	39.3%
	Q2 (26–50%)	71	14.8%	32	12.2%
	Q3 (51–75%)	110	22.9%	58	22.1%
	Q4 (76–100%)	114	23.7%	69	26.3%
Work characteristics					
Physical load	1–5	1.76	0.86	1.60	0.77
Mental load	1–5	4.18	0.67	4.20	0.64
Procedural justice	1–5	3.28	0.81	3.36	0.84
Working in (semi-)skilled manual labor	Yes	50	6.8%	22	5.7%
Working in transport	Yes	17	2.3%	11	2.8%
Working in healthcare	Yes	101	13.7%	44	11.4%
Permanent contract	Yes	671	91.0%	355	92.7%
Supervisory tasks	Yes	172	23.4%	114	29.6%
Working with substances	Yes	168	22.7%	71	18.4%
Evening / night shifts	Yes	261	35.5%	148	38.3%
Years of physically heavy work	> = 8 years	263	35.9%	97	25.3%
Years of mentally heavy work	> = 16.5 years	356	48.9%	184	48.3%
Health					
Perceived health	Good	551	74.6%	^a^	^a^
Perceived physical health	Average 50	47.64	9.22	55.26	4.08
Feeling full of life	Yes	564	76.6%	354	91.9%
Feeling worn out	No	636	86.3%	371	96.4%
Fatigue	No	555	75.4%	365	94.8%
Depressive symptoms	1–4	1.53	0.46	1.35	0.34
Recovery and relaxation	1–5	2.99	0.76	3.06	0.75
Problems with leg / feet	No	309	42.6%	287	75.9%
Body height	Centimeters	174.26	8.97	175.39	9.43
BMI	Kg / m^2^	27.82	4.64	25.67	3.42
# days active for at least 30 min / week	> = 2 days	553	75.0%	298	77.4%
# days intensively active for at least 20 min / week	> = 2 days	282	38.5%	161	41.9%
Social factors					
Partner with paid employment	Yes	239	32.5%	140	36.4%
Work disabled partner	Yes	40	5.4%	21	5.5%
Attitude partner with regard to quitting work	No partner	191	26.2%	77	20.4%
	(Very) unpleasant	42	5.8%	29	7.7%
	Neither pleasant nor unpleasant / pleasant	497	68.1%	271	71.9%
Attitude partner with regard to continued employment	No partner	191	26.5	77	20.5%
	Very unpleasant	53	7.4%	24	6.4%
	Unpleasant	94	13.0%	49	13.0%
	Neither pleasant nor unpleasant	258	35.8%	131	34.8%
	Pleasant	91	12.6%	67	17.8%
	Very pleasant	34	4.7%	28	7.4%
Missing family activities due to work obligations	Yes	346	46.8%	164	42.5%

*Q* quartile. ^a^These numbers were below the privacy-based norm of Statistics Netherlands and may not be shown

### Employees with a chronic disease

The domain analyses within the group of employees with chronic disease showed that the domains of demographic, socioeconomic and work characteristics, health, and social factors contained significant predictors of working beyond retirement (Table 2). The area under the curve ranged from 0.56 to 0.65, which indicates a poor accuracy of the prediction models.

**Table 2:** Logistic regression analyses, domain analyses: prediction of working beyond retirement among employees with and without chronic disease

	Unit, categories, range	Employees with chronic disease (N = 739)		Employees without chronic disease (N = 386)	
		OR	95% CI	OR	95% CI
Demographic characteristics					
Educational level	Low	1	Reference		
	Intermediate	1.13	0.72–1.78		
	High	1.73*	1.13–2.66		
Nagelkerke R^2^		0.02			
Area under the curve		0.56	0.51–0.61		
Socioeconomic characteristics					
Option value	Ln	0.91*	0.86–0.97		
Quartile income	Q1 (0–25%)	1	Reference		
	Q2 (26–50%)	1.42*	0.64–3.18		
	Q3 (51–75%)	1.49*	0.72–3.09		
	Q4 (76–100%)	2.26*	1.08–4.74		
Quartile equity	Q1 (0–25%)	1	Reference		
	Q2 (26–50%)	0.93	0.55–1.58		
	Q3 (51–75%)	0.62*	0.39–0.99		
	Q4 (76–100%)	0.58*	0.35–0.96		
Nagelkerke R^2^		0.08			
Area under the curve		0.65	0.59–0.71		
Work characteristics					
Physical load	1–5	0.70*	0.56–0.89		
Mental load	1–5	1.40*	1.05–1.87		
Working in (semi-)skilled manual labor	Yes			0.10*	0.01–0.77
Working in transport	Yes	2.69*	0.92–7.91		
Working in healthcare	Yes	1.84*	1.14–2.99		
Permanent contract	Yes	0.34*	0.19–1.95		
Evening / night shifts	Yes	1.34*	0.92–1.87	1.43*	0.90–2.27
Years of physically heavy work	> = 8 years			1.54*	0.91–2.59
Nagelkerke R^2^		0.08		0.05	
Area under the curve		0.65	0.60–0.70	0.60	0.54–0.66
Health					
Perceived health	Good	0.54*	0.31–0.96		
Perceived physical health	Average 50	1.05*	1.02–1.08	1.08*	1.00–1.17
Feeling full of life	Yes			7.40*	1.34–41.05
Feeling worn out	No	2.11*	0.91–4.88	0.19*	0.03–1.11
Depressive symptoms	1–4	0.56*	0.31–1.02		
Recovery and relaxation	1–5			0.61*	0.43–0.86
Body height	Centimeters	1.03*	1.01–1.05	1.02	1.00–1.05
# days intensively active for at least 20 min / week	> = 2 days			1.78*	1.09–2.90
Nagelkerke R^2^		0.08		0.11	
Area under the curve		0.65	0.60–0.70	0.67	0.62–0.73
Social factors					
Attitude partner with regard to continued employment	No partner	1	Reference	1	Reference
	Very unpleasant	0.07*	0.01–0.50	0.47	0.14–1.53
	Unpleasant	0.67	0.35–1.29	0.15*	0.04–0.54
	Neither pleasant nor unpleasant	1.53*	0.99–2.37	0.89	0.48–1.66
	Pleasant	1.23	0.69–2.22	2.02*	1.02–4.01
	Very pleasant	0.93	0.38–2.28	0.51	0.17–1.51
Missing family activities due to work obligations	Yes	0.68*	0.48–0.98		
Nagelkerke R^2^		0.07		0.11	
Area under the curve		0.62	0.57–0.66	0.66	0.60–0.72

*Q* quartile, *p value< 0.15

In the final model (Table 3), the domains of work and health remained statistically significant. Of the work characteristics, working in healthcare compared to working in other sectors predicted working beyond retirement. Employees having a permanent contract before retirement were less likely to work beyond retirement. Those with higher physical workload were less likely to work beyond retirement. Of the health domain, better perceived physical health and higher body height predicted working beyond retirement. The area under the curve of the final model for retirees with a chronic disease was 0.66, which indicates a poor accuracy of the prediction model. Nagelkerke’s pseudo R2 was low (0.10).

**Table 3:** Logistic regression analyses, final model: prediction of working beyond retirement among employees with and without chronic disease

	Unit, categories, range	Employees with chronic disease (N = 739)		Employees without chronic disease (N = 386)	
		OR	95% CI	OR	95% CI
Work characteristics					
Physical load	1–5	0.72	0.56–0.92		
Working in (semi-)skilled manual labor	Yes			0.10	0.01–0.75
Working in healthcare	Yes	2.11	1.26–3.54		
Permanent contract	Yes	0.33	0.19–0.57		
Health					
Perceived physical health	Average 50	1.04	1.02–1.07		
Feeling full of life	Yes			5.35	1.21–23.60
Recovery and relaxation	1–5			0.63	0.44–0.90
Body height	Centimeters	1.03	1.01–1.06		
# days intensively active for at least 20 min / week	> = 2 days			2.36	1.40–3.98
Social factors					
Attitude partner with regard to continued employment	No partner			1	Reference
	Very unpleasant			0.40	0.12–1.36
	Unpleasant			0.11	0.03–0.39
	Neither pleasant nor unpleasant			0.81	0.42–1.57
	Pleasant			1.74	0.85–3.58
	Very pleasant			0.33	0.10–1.11
Nagelkerke R^2^		0.10		0.22	
Area under the curve		0.66	0.62–0.71	0.75	0.70–0.80

### Employees without a chronic disease

The domain analyses within the group of employees without chronic disease showed that the domains of work characteristics, health and social factors contained significant predictors of working beyond retirement (Table 2). The area under the curve of these domains ranged from 0.60–0.67, which indicates a poor accuracy of the prediction models.

All three domains remained statistically significant in the final model (Table 3). Of the work characteristics, those who worked in (semi-)skilled manual labor were less likely to work beyond retirement compared to those working in other sectors. From the health domain, feeling full of life and being intensively physically active for at least 2 days per week predicted working beyond retirement, whereas those with better recovery worked beyond retirement less often. Of the social factors, those with a partner who did not like them to work until the statutory retirement age worked beyond retirement less often compared to those without a partner. The area under the curve of the final model for retirees without a chronic disease was 0.75, which indicates a fair degree of accuracy of the prediction model. Nagelkerke’s pseudo R2 was relatively low (0.22).

### Similarities and differences between employees with and without a chronic disease

The analyses showed that the domains of work and health contained significant predictors of working beyond retirement both in employees with and without a chronic disease. Although the domains of demographic and socioeconomic characteristics contained significant predictors of working beyond retirement in employees with a chronic disease in the domain analyses, this was not the case in employees without a chronic disease. In the final models, these domains were not statistically significant in both groups. The social domain only contained significant predictors of working beyond retirement among employees without a chronic disease, and not among employees with a chronic disease.

## Discussion

This study showed that work and health factors independently predicted working beyond retirement in employees with and without a chronic disease, whereas social factors only independently contributed to the prediction of working beyond retirement among employees without a chronic disease. Demographic and socioeconomic characteristics did not independently contribute to the prediction of working beyond retirement in any group, in addition to the other domains. Based on the prediction model properties, it seems more difficult to predict working beyond retirement among employees with than those without a chronic disease. But in general, despite inclusion of multiple potential predictors in the domains of work characteristics, health, social factors and socioeconomic factors, it was difficult to accurately predict working beyond retirement for employees with and without chronic disease.

Our previous study on the prediction of working beyond retirement showed that work characteristics, health and social factors predicted working beyond retirement [3]. This study, and (to the knowledge of the authors) also other studies on working beyond retirement, did not take into account that prediction of working beyond retirement may be different for workers with and without chronic diseases. By doing so, the present study is an extension of our previous study as well as an important contribution to the field. Previous research on determinants of workforce participation and early exit from the labor market provided indications that older workers with chronic diseases have specific needs when it comes to participation in paid work [11, 24]. However, in our study, two of the five domains of working beyond retirement were relevant for employees with and without a chronic disease. As this study showed that work and health factors play a role both in employees with and without a chronic disease, it might be that on a domain level, there are no big differences, but that specific needs and considerations for workers with a chronic disease, that are different from the group without a chronic disease, may take place on a domain-specific level. This study showed that domain-specific predictors of working beyond retirement varied between employees with and without a chronic disease, which is a novel and important contribution to the understanding of working beyond retirement. Another possible explanation might be that the group with a chronic disease was a healthy selection of the total population with chronic diseases, as they had managed to continue their participation in paid work until older age, i.e. a healthy worker effect [25]. This group may have settled their health problems or already adjusted their work environment in such a way that it fits the limitations resulting from a disease. On the other hand, the group without a chronic disease may have other health problems resulting in limitations in work that are comparable to the group with a chronic disease. Furthermore, this study showed that social factors independently contributed to the prediction of working beyond retirement among employees without a chronic disease and not among employees with a chronic disease. Seemingly, work and health factors outweigh the role of social factors when it comes to working beyond retirement in employees with a chronic disease, which is a novel finding that contributes to the field of research. As far as the authors know, no previous studies investigated the role of social factors specifically for workers with and without chronic diseases.

Because predicting work beyond retirement was more difficult among employees with a chronic disease than among employees without a chronic disease, there may be additional factors among chronically ill employees which were not included in the study. Additional factors may lie within the domain of work characteristics. Previous research showed for example that employees with chronic diseases benefited more from psychosocial resources at work (e.g. social support and autonomy) than employees without chronic diseases [11]. Another study supported the idea that autonomy may be especially important for continued participation in work, as autonomy buffered the adverse effect of having a chronic disease on early exit from the workforce [12]. These variables were investigated in our previous study [3], but did not predict working beyond retirement in the total population, and therefore they were not included in the present study. Another explanation for the lower predictive ability may be that the group with chronic diseases is more heterogeneous than the group without chronic diseases. A previous study, for example, showed that there are differences between chronic diseases when it comes to loss of paid employment [24]. Having psychological and musculoskeletal health problems predicted early retirement, whereas other chronic diseases did not. Next to the fact that the type of chronic disease influences whether someone continues working or not, predictors of working beyond retirement may also be different among chronic diseases. To illustrate, someone with psychological health problems benefits from lower mental demands, whereas someone with musculoskeletal health problems rather benefits from lower physical demands. Future research is needed to investigate whether additional predictors play a role among employees with a chronic disease and whether different chronic diseases have a different etiology of working beyond retirement. Especially, explorative qualitative research could be helpful to discover additional predictors and necessary conditions to prolong their working lives for employees with a chronic disease.

The main strength of this study is that we linked questionnaire data, which is the most common data source for public health research, to register data, which is the most common used data source for research in economics, to investigate predictors of working beyond retirement among employees with and without a chronic disease. In addition, we used longitudinal data, meaning that independent variables were measured at an earlier moment in time than working beyond retirement. This allowed us to investigate predictors instead of associations. It is likely, however, that predictors changed over time, which we did not take into account. This might be a good suggestion for future research on working beyond retirement. Another limitation is that we were not able to do separate analyses for different chronic diseases, whereas there may be differences between chronic diseases. Future research with larger sample sizes is needed to explore differences in predictors of working beyond retirement between various chronic diseases. Furthermore, we did not distinguish between working beyond early and statutory retirement, as the sample size did not allow us to separate between these two types. However, as a previous study showed that determinants of working beyond early and statutory retirement do not really differentiate [4], we do not expect major bias from this. Another limitation is that we used a self-reported measure of chronic disease. As participants were not asked whether the disease was previously diagnosed by a health professional, and there was no additional qualifying information with regard to the duration of the disease, the answers among participants may deviate from the judgment of a health professional. Furthermore, almost every employee without a chronic disease reported good perceived health. Thus, this factor did not show any contrast in this group. However, to allow comparison with our previous study [3] we chose to keep this cut-off value. It might be good to use a different cut-off value that distinguishes between those with better and poorer perceived health in future research. In addition, no distinction was made between employees with one chronic disease as opposed to employees with multi-morbidity. Future research is needed to discover whether this is an important distinction when it comes to working beyond retirement.

## Conclusions

In conclusion, this study showed that work and health factors independently predicted working beyond retirement in employees with and without a chronic disease, whereas social factors only independently contributed to the prediction of working beyond retirement among employees without a chronic disease. This indicates that underlying mechanisms of working beyond retirement may be different for the large group of older workers with a chronic disease. Predicting working beyond retirement among employees with chronic diseases was more difficult than among those without chronic diseases. Therefore, future research is needed to investigate which additional factors may play a role in employees with chronic diseases, and whether there are differences in working beyond retirement between different chronic diseases.
